# Supplementary material for: ‘The broker also told me that I will not have problems after selling because we have two and we can survive on one kidney’: Findings from an ethnographic study of a village with one kidney in Central Nepal
Source: PLOS Glob Public Health. 2022 Oct 14;2(10):e0000585. doi: 10.1371/journal.pgph.0000585 (PMC10021627; doi:10.1371/journal.pgph.0000585)
Supplement: S3 File — (DOCX) [file pgph.0000585.s003.docx]

# *‘The broker also told me that I will not have problems after selling because we have two and we can survive on one kidney’*: findings from an ethnographic study of a village with one kidney in Central Nepal

Bijaya Shrestha^1*^, Bipin Adhikari^2,3^, Manash Shrestha^1^, Ankit Poudel^5^, Binita Shrestha^6^ Dev Ram Sunuwar^7^, Shiva Raj Mishra^8,9^, Luechai Sringernyuang^1,4*^

^1^Department of Society and Health, Faculty of Social Sciences and Humanities, Mahidol University, Nakhon Pathom, Thailand

^2^Mahidol-Oxford Tropical Medicine Research Unit, Faculty of Tropical Medicine, Mahidol University, Bangkok, Thailand

^3^Centre for Tropical Medicine and Global Health, Nuffield Department of Medicine, University of Oxford, Oxford, UK

^4^Contemplative Education Center, Mahidol University, Nakhon Pathom, Thailand

^5^Independent researcher, Bharatpur-05, Chitwan, Nepal

^6^Independent researcher, Tanahun-05, Nepal

^7^Department of Nutrition and Dietetics, Armed Police Force Hospital, Kathmandu, Nepal

^8^Melbourne School of Population and Global Health, University of Melbourne, Australia

^9^ Academy for Data Sciences and Global Health, Kathmandu, Nepal

*Corresponding author

Luechai Sringernyuang, Ph.D.

Department of Society and Health, Faculty of Social Sciences and Humanities,

Mahidol University, 999 Phuttamonthon 4 Road, Salaya, Nakhon Pathom 73170 Thailand

Email: luechaisri@gmail.com

Telephone Number: +66-81-934-8454

| **Codes: Child** | **Code: Parents** | **Description** | **Themes** |
| --- | --- | --- | --- |
| Daily wage workers | Poor socio-economic status | It means the economic status of the participants, therefore, they had to perform different activities and they are marginalized in the society. | Vulnerability |
| Poverty (14) |  |  |  |
| Dependent on donation from NGO |  |  |  |
| Manual work for sellers |  |  |  |
| Lack of employment (3) |  |  |  |
| Thought will earn huge money |  |  |  |
| No proper irrigation facilities (2) |  |  |  |
| Lack of food supplies (2) |  |  |  |
| Working in India |  |  |  |
| Alcoholic (9) | Alcohol Drinking | It is the behavior of the participant about drinking habit and their culture. It is the place where they meet the broker and sellers. |  |
| Detoriated health condition |  |  |  |
| Drinking culture (7) |  |  |  |
| Broker giving idea of kidney selling |  |  |  |
| Relieves pain if we drink (3) |  |  |  |
| Lack of knowledge about kidney and it work (8) | Ignorance/gullibility | It means they are naïve and they are unaware and uneducated. They do not have knowledge about kidney and its use. |  |
| Drinking to subside pain (4) |  |  |  |
| Trapped by the story of better future (8) |  |  |  |
| Believe the false news (2) |  |  |  |
| Feels stigmatized because of selling (10) |  |  |  |
| Follow the caste system and discrimination (3) |  |  |  |
| Do not know what is kidney |  |  |  |
| Lavish life and follow all the neighbor after selling kidney | Social conformity | It means, they are following the culture set by others. |  |
| Everyone did in neighbor (4) |  |  |  |
| Destroyed their house due to earthquake | Indirect incentives | It is the incentive provided to the sellers through economy, false hope and wrong information. |  |
| Wrong information about donation |  |  |  |
| Donation received from foreign donors (3) |  |  |  |
| Proximity to cities (3) | Shifting of business | It is the issue where the business is shifted to new place due to the booming business in other place and lack of business in one place due to change of roadways. | Contextual factors |
| Migration from village (3) |  |  |  |
| Change of business destination |  |  |  |
| Loss of Job opportunities |  |  |  |
| No care after operation | Role of medical personnel | It is about the role of medical personal throughout the kidney transplantation. |  |
| Role of doctors to approve for operation |  |  |  |
| No information shared by doctors |  |  |  |
| False hope | Fake documentations | It is the preparation of the document during the kidney selling process. |  |
| fake papers arranged (5) |  |  |  |
| wrong name used |  |  |  |
| Making stamp and papers (3) |  |  |  |
| No policy to address kidney trafficking (6) | Policy loopholes | It is the loopholes in the rules and regulation of the country where they can utilize the policy in their benefits. |  |
| Open border (2) |  |  |  |
| Consent was enough |  |  |  |
| No proper work form NGO or legal authorities (2) |  |  |  |
| kidney sellers are culprit (2) |  |  |  |
| Signed document easily prepared with stamps |  |  |  |
| Birth of broker | It is the role of brokers during the kidney selling situation. | | Role of a broker |
| lured by money |  |  |  |
| Taught about the kidney and its quantity |  |  |  |
| Did not give promised money |  |  |  |
| Meeting brokers |  |  |  |
| Ways of brokers being friend |  |  |  |
| Taught the way of earning money |  |  |  |
| Wrong information |  |  |  |
| Fake donors prepared |  |  |  |
| Coordination and management from pick up to operation (3) |  |  |  |
| Convinced by brokers (9) |  |  |  |
| Fraud by broker (9) |  |  |  |
| Multiple roles by brokers (6) |  |  |  |
