# Supplementary material for: ‘The broker also told me that I will not have problems after selling because we have two and we can survive on one kidney’: Findings from an ethnographic study of a village with one kidney in Central Nepal
Source: PLOS Glob Public Health. 2022 Oct 14;2(10):e0000585. doi: 10.1371/journal.pgph.0000585 (PMC10021627; doi:10.1371/journal.pgph.0000585)
Supplement: Attachment — Submitted filename: RESPONSE TO REVIEWERS COMMENTS.docx [file pgph.0000585.s004.docx]

Date: 14^th^ May 2022

Dear editor and reviewers,

Thank you very much for your detailed and constructive evaluation; and opportunity to revise our work. We have followed each of your suggestions to improve the manuscript. Please see below our responses to your specific suggestions with corresponding changes in the manuscript.

We look forward to your kind consideration.

Sincerely,

Bijay Shrestha

1. Please provide an Author Summary. This should appear in your manuscript between the Abstract (if applicable) and the Introduction, and should be 150–200 words long. The aim should be to make your findings accessible to a wide audience that includes both scientists and non-scientists. Sample summaries can be found on our website under Submission Guidelines:

<https://journals.plos.org/globalpublichealth/s/submission-guidelines#loc-parts-of-a-submission>
AUTHORS: Thank you for your suggestion. We have added an author summary in the manuscript and reads as follows:

**Author summary**

Kidney sale is a global phenomenon shaped by poverty and poor governance in low-income nations, with higher-income countries serving as beneficiaries and lower-income countries serving as donors. Over the years, a rising number of individuals in Hokse village near Nepal's capital city have sold their kidneys. Using a combination of methods including ethnography and multi-stakeholder consultations, this research aimed to investigate the motivations for kidney sale and their repercussions. Reasons for kidney selling ranged from individual’s vulnerability, to wider social, and contextual factors, among which role of brokers were critical. Although poverty remains to be a common motivation for kidney selling in Hokse, brokers played pivotal role in exploiting the poverty and vulnerability of the community members, thereby making Hokse unique to other poverty striken villages where kidney selling is uncommon. There is an urgent need to strengthen the policy and regulation to curb the strategies and methods exploited by brokers in Hokse village before it continues to spread outside the village.
2. Please upload all main figures as separate Figure files in .tif or .eps format only. For more information about how to convert and format your figure files please see our guidelines:

<https://journals.plos.org/globalpublichealth/s/figures>

AUTHORS: Thank you for your suggestion. We have revised the figure files and have uploaded the figures in tif format.

3. All figures and supporting information files will be published under the Creative Commons Attribution License ([creativecommons.org/licenses/by/4.0/](http://creativecommons.org/licenses/by/4.0/)). Authors retain ownership of the copyright for their article and are responsible for third-party content used in the article.

Figure 1: please (a) provide a direct link to the base layer of the map used and ensure this is also included in the figure legend; (b) provide a link to the terms of use / license information for the base layer. We cannot publish proprietary or copyrighted maps (e.g. Google Maps, Mapquest) and the terms of use for your map base layer must be compatible with our CC-BY 4.0 license.

AUTHORS: Thank you for your suggestion. Please see below the figure legend that responds to your queries ‘a’ and ‘b’. We have provided the link for base layer (shape files) and they are publicly available for unrestricted use.

Figure 1: The study site is selected Hokse village, ward numbers 7 and 8 of Paanchkhal Municipality, Kavrepalanchok district, Nepal. The map was created using ArcGIS desktop version 10.8. The shapefile of the administrative districts and location for Nepal was obtained from the Government of Nepal, Ministry of Land Management, Survey Department website and were publicly available for unrestricted use (<http://www.dos.gov.np/nepal-map>).

PGPH-D-22-00320

Additional Editor Comments (if provided):

I received two external reviews for this manuscript. Both reviewers were generally positive but have suggestions to improve the manuscript. Please review these suggestions and provide a detailed point by point response.

In addition, I have additional comments:


1. The data sharing statement doesn't adhere to the journal philosophy of open data access. Although it is true that qualitative data is often identifiable and can't be directly shared, the authors can prepare a more complete digest of the data for sharing. This should at least include the full code book used and a table of coding frequencies. This can be done without violating any confidentiality concerns.

AUTHORS: Thank you for your suggestion. We have added a data availability statement in the manuscript and reads as follows:

**Availability of data and material**

Data cannot be shared publicly because of the nature of the data being qualitative that contains personal quotes and clues to where the study occurred and can be potentially identifiable. However, data is available on request to the chair of the Academy for Data Sciences and Global Health (ADSGH), Kathmandu, Nepal (E-mail: shivarajmishra@gmail.com) complying with the data access policy by Institutional Review Committee (IRC) of Nepal Health Research Council and ADSGH.

2. The Discussion largely repeats the Results, leading to a lot of redundancy and not a lot of added value. The discussion should be distinct from the results, and it could be more synthetic and more engaged with international literature and with policy implications.

AUTHORS: Thank you very much for your suggestion. We have revised the ‘Discussion’ section mostly to make it succinct, retain important points and relevant with the international literature. We have also added the final policy implications in the revised ‘conclusion’ section.

3. Although the background of the work is broadly ethnographic, the bulk of what is presented here is focused interviews with thematic coding. As such (and in line with journal policy) a checklist should be used to make sure all elements of the research design and methods are reported. Please use an EQUATOR recommended checklist to check and revise the paper (<https://www.equator-network.org/reporting-guidelines-study-design/qualitative-research/>)

AUTHORS: Thank you for your concern. We agree with your suggestion. We have used a COREQ checklist (revised under ‘study design and participants’) for this study and has been added as an ‘appendix A’ in this study.

# Title: *‘The broker also told me that I will not have problems after selling because we have two and we can survive on one kidney’*: findings from an ethnographic study of a village with one kidney in Central Nepal

**Reviewer #1**

Reviewer #1: The manuscript is very well structured. The details that explain and describe the ethnographic approach/methodology are technically sound; and the theoretical framework references key publications on the topic of global organ trafficking in humans. The findings section includes a graphic illustration of the interaction between vulnerability and contextual factors that influence individual and community-level mindsets and experiences.
AUTHORS: Thank you for your comprehensive evaluation.
Minor edits suggested to the authors:

(a) Results section: Specify dates for fieldwork and data collection. These details are particularly relevant to better understand the significance of past events like the earthquake.
AUTHORS: Thank you for the suggestion related to specifying the dates for the fieldwork for clearer picture to understand the events like earthquake. We have added the dates under the section ‘data collection’ and reads as follows:

The fieldwork started in February 2021 and the data analysis was completed in September 2021.

(b) Discussion section - overview: Consider adding a couple of sentences on the profile and mindset of the informant who was a seller and became a broker (in the results section), to better display the motivations/justifications behind liaising in the system in the discussion.

AUTHORS: Thank you for the suggestion. We have added the profile of the seller who became the broker in both result and discussion section and reads as follows:

**Results**

***Participant characteristics***

One of the kidney seller who later became broker was working as a chef in an Indian restaurant who realized an opportunity to make an extra money by exploiting the familiarity and trust he had in his own village.

**Discussion**

***Overview***

One of the prominent reasons how kidney sellers later became brokers was because of their existing relationship and trust in their community where they saw an opportunity to generate an easy money.

(c) Figure 3: Poverty due to earthquake is mentioned as relevant in the discussion, but it is not included as a village/community factor in Figure 3 (i.e., vulnerability due to natural disasters or adverse events).

AUTHORS: Thank you for specifying the ‘poverty due to earthqauke’. Largely, we agree that ‘poverty’ is the major factor contributing to the dynamics of kidney selling in the community. Indeed poverty due to earthquake therefore does contribute to the existing economic condition of the community and the residents. Nonetheless, we believe that poverty is not only the factor for kidney selling. As there are equally poorer communities in Nepal but they are not engaged in kidney selling. In fact, based on our ethnographic findings, we found the role of brokers to be pivotal, thus we have highlighed the issue of brokerage as major factor for kidney selling in figure 3.

(d) Conclusión: Specify a couple of examples of the most urgent “regulatory measured” that can contribute to curb the kidney market, based on the arguments presented in the discussion section.

AUTHORS: Thank you for suggestions regarding the urgent “regulatory measures” to tackle the situation of kidney selling. We have revised the ‘conclusion’ section to address the concerns and reads as follows:

Although increasing support for livelihood and education is critical, regulatory measures are urgently needed to curb the kidney trade. First, regulatory authorities should mandate a thorough background checking of a donor such as cross-check with their family members, local authorities, and legal representatives. Second, facilitator can always be a broker disguising as a family member and therefore needs to be suspected and explored for his/her potential role as a motivator. Third, cross-border collaboration between Nepal and India needs to scrutinize the legitimacy of consent process, documents, and potential involvement of a broker.

**Reviewer #2**

Reviewer #2: The authors have done a good job in gathering and presenting the data on which the paper is based. The content of the discussion section follows logically from the authors' analysis of the factors behind the high rate of kidney selling in the village.

However, mysteriously missing from the paper is any reference to time. When was all this selling taking place? The authors say for decades. If for decades, why have the villagers not learned over time that the promises of the brokers that selling a kidney would not affect their health were not true? If about one third of villagers have sold a kidney, surely some have suffered medical complications. Has the rate of selling remained the same over the "decades" or gone up or down over time? Why? The authors indicate that bibliographic reference #28 provides more information regarding their methodology, but it is unclear what this reference is. What is the name of this journal?

AUTHORS:Thank you for your evaluation and concerns. We agree with your point about missing the reference time. We have revised and added the statement within the Results section and reads as follows:

In the village, most of the kidney selling occurred between early 1990s and 2000, the kidney sellers interviewed in this study were in their mid-20s to 30s when they sold their kidneys.

To detail firther here, this research was conducted in 2021 but most of the kindey selling occured between early 1990 and 2000, nonetheless, we do not have exact time frame of kidney selling and was beyond the scope of this study. According to our study findings, most of the villagers were motivated by economic incentives, persuation by brokers and social conformism. Community members tended to follow their fellow neighbors and thus was quite concentrated in the village. Although much less common now (also because most who would, have already sold their kidneys), it contines to affect the village with medical complications and issues related to economic sustainabilities. Such complications may have deterred few villagers from selling, although it did not come up in our data and analysis.

The article #28 is available in Journal of Medical Internet Research (Research Protocol). The details are available on: [doi:10.2196/29364](https://doi.org/10.2196/29364)

Table 2 presents characteristics of the 10 kidney sellers. They range in age from the early 40s to 60. Presumably this is their age at the time of the study not at the time of their involvement in the trade. How old were they when they actually sold their kidney? What specifically did they need the money for? The gender ratio of men to women in the sample is 7:3. Is this ratio typical of the much larger population of sellers in the village? The authors state that altruistic kidney donors were not included in the study. But how do they know who was or was not an altruistic donor? All of the documentation that would-be donor/sellers fill out is to demonstrate that they are kin or altruistic donors since commercial sale is illegal. In fact, one of the most interesting quotes that the authors include is from a transplant surgeon who seemingly bemoans the fact that medical personnel are essentially compelled to carry out transplants when the paperwork is correctly (however falsely) filled out.

AUTHORS: Thank you very much for your useful questions. We have revised to address your concerns in ‘result’ section and reads as follows:

A total of 43 participants were enrolled in this study, including nine kidney sellers and one kidney seller who was also a broker (Tables 1 and 2). In the village, most of the kidney selling occurred between early 1990 and 2000, the kidney sellers interviewed in this study were in their mid-20s to 30s when they sold their kidneys. Higher proportion of males were engaged in kidney selling compared to females (Male versus female: 7:3) and it accurately reflects the kidney sellers’ population in the village.

Since we carried out an ethnographic study to explore the drivers of kidney selling in the village, it is based on our multi-methods approach that we identified their motivations behind kidney selling. In addition, since the kidney selling already occurred, sharing the reasons for kidney selling was not legally problematic and therefore, we were able to know how they pretended to be altruistic donors when they approached transplant surgeon.

Indeed, it is a painful dilemma for a transplant surgeon who could suspect the fake documents, but was compelled to abide by the completed documents (written informed consent), and responses from the donors and recipients. Thus we have explicitly revised the conclusion to address these loopholes and reads as follows:

First, regulatory authorities should mandate a thorough background check of a donor such as cross-check with their family members, local authorities, and legal representatives. Second, facilitator can always be a broker disguising as a family member and therefore needs to be suspected and explored for his/her potential role as a motivator. Third, cross-border collaboration between Nepal and India needs to scrutinize the legitimacy of consent process, documents, and potential involvement of a broker.

The authors make the important point that selling a kidney across borders complicates legal issues if something goes wrong with the transaction. This would certainly make cross border transaction more attractive to the brokers and buyers. Does that imply that selling occurs only or primarily as a cross-border phenomenon? Do the villagers take part in any commercial transactions within Nepal?

AUTHORS: Thank you for these interesting questions regarding the cross-border transaction. I think kidney sellers would be aware of and suspect cross-border transaction complications. So, to avoid any last-minute changes in plans, based on our study, most kidney sellers would receive installments and persuasions to adequately gain the trust. Nonetheless, we cannot disagree with you that brokers can always deceive kidney sellers once the organ donation is completed and is easier in India. Also, to note is, during the most kidney sellings that occurred in Hokse, kidney transplant was not yet available in Nepal. But yes, over the years, kidney transplant is occurring in Nepal, there are few reports of kidney sellings (transplants) from Nepal. Nonetheless majority of illegal transplantation still occurs in India.

In their concluding sentence the authors state "regulatory measures are urgently needed to curb the kidney trade", but their paper has emphasized the total failure of the current regulatory regime to play any meaningful role in curbing the trade. What concrete regulatory changes do they see actually likely to have an impact on the organ trade?

AUTHORS: Thank you very much for your kind suggestions. We have revised the conclusion section to offer concrete solutions to curb the current regulatory failures. The revised statements read as follows:

First, regulatory authorities should mandate a thorough background check of a donor such as cross-check with their family members, local authorities, and legal representatives. Second, facilitator can always be a broker disguising as a family member and therefore needs to be suspected and explored for his/her potential role as a motivator. Third, cross-border collaboration between Nepal and India needs to scrutinize the legitimacy of consent process, documents, and potential involvement of a broker.

Finally, while generally well-written, there are places where copy editing would be useful. The authors should have a non-specialist reader go through the paper.

AUTHORS: Thank you for very much for the suggestion. We had our manuscript copy-edited by a native English speaker.
